# Absolute numbers of lives saved and overdiagnosis in breast cancer screening, from a randomized trial and from the Breast Screening Programme in England

**DOI:** 10.1258/jms.2009.009094

**Published:** 2010-03

**Authors:** Stephen W Duffy, Laszlo Tabar, Anne Helene Olsen, Bedrich Vitak, Prue C Allgood, Tony H H Chen, Amy M F Yen, Robert A Smith

**Affiliations:** CR-UK Centre for Epidemiology, Statistics and Mathematics, Wolfson Institute for Preventive Medicine, Barts and the London School of Medicine and Dentistry, Queen Mary University of London, Charterhouse Square, London EC1M 6BQ, UK; University School of Medicine, Uppsala, SwedenDepartment of Mammography, Central Hospital, Falun, Sweden; CR-UK Centre for Epidemiology, Statistics and Mathematics, Wolfson Institute for Preventive Medicine, Barts and the London School of Medicine and Dentistry, Queen Mary University of London, Charterhouse Square, London EC1M 6BQ, UK; Department of Mammography, University Hospital, Linköping, Sweden; CR-UK Centre for Epidemiology, Statistics and Mathematics, Wolfson Institute for Preventive Medicine, Barts and the London School of Medicine and Dentistry, Queen Mary University of London, Charterhouse Square, London EC1M 6BQ, UK; Division of Biostatistics/Centre of Biostatistics Consultation, Graduate Institute of Epidemiology, College of Public Health, National Taiwan University, Taipei, Taiwan, ROC; Division of Biostatistics/Centre of Biostatistics Consultation, Graduate Institute of Epidemiology, College of Public Health, National Taiwan University, Taipei, Taiwan, ROC; Department of Cancer Control Science, American Cancer Society, Atlanta, GA, USA

## Abstract

**Objectives:**

To estimate the absolute numbers of breast cancer deaths prevented and the absolute numbers of tumours overdiagnosed in mammographic screening for breast cancer at ages 50–69 years.

**Setting:**

The Swedish Two-County randomized trial of mammographic screening for breast cancer, and the UK Breast Screening Programme in England, ages 50–69 years.

**Methods:**

We estimated the absolute numbers of deaths avoided and additional cases diagnosed in the study group (active study population) of the Swedish Two-County Trial, by comparison with the control group (passive study population). We estimated the same quantities for the mortality and incidence rates in England (1974–2004 and 1974–2003, respectively). We used Poisson regression for statistical inference.

**Results:**

A substantial and significant reduction in breast cancer mortality was associated with screening in both the Two-County Trial (*P* < 0.001) and the screening programme in England (*P* < 0.001). The absolute benefits were estimated as 8.8 and 5.7 breast cancer deaths prevented per 1000 women screened for 20 years starting at age 50 from the Two-County Trial and screening programme in England, respectively. The corresponding estimated numbers of cases overdiagnosed per 1000 women screened for 20 years were, respectively, 4.3 and 2.3 per 1000.

**Conclusions:**

The benefit of mammographic screening in terms of lives saved is greater in absolute terms than the harm in terms of overdiagnosis. Between 2 and 2.5 lives are saved for every overdiagnosed case.

## INTRODUCTION

Estimation of the absolute benefits and harms of mammographic screening for breast cancer remains a subject of discussion. The estimates from individual randomized trials and service screening programmes suggest that between 300 and 500 women need to be screened every 2–3 years for 10 years to prevent one death from breast cancer.^1,2^ Harms of screening include the anxiety and inconvenience associated with screening and with suspicious screening findings, which do not result in a diagnosis of cancer, and overdiagnosis, the last of which has attracted considerable interest recently.^3–6^

Overdiagnosis is defined as the diagnosis of a cancer as a result of screening that would not have been diagnosed in the woman's lifetime had screening not taken place. Estimates of overdiagnosis range from less than 10% of tumours diagnosed in a screening programme to around 50%.^3,4,7,8^

There is a majority of view that the benefits of breast screening outweigh the harms,^9,10^ although debate on target age ranges remains.^11^ Recently, however, it has been claimed that the benefits in terms of lives saved are much smaller, and the harm in terms of overdiagnosis much larger, than had previously been thought. Gøtzsche *et al.*^6^ have asserted that for 2000 women screened for 10 years, only one life will be saved and six additional cases will be diagnosed. The accuracy of these claims has been questioned, however.^12^ It is therefore worthwhile to seek estimates from randomized trials and service screening to confirm or refute these claims.

The Swedish Two-County Trial was the first published randomized trial of breast screening using mammography as the sole screening modality.^13^ Its primary result was a 30% reduction in breast cancer mortality with the offer of screening. Its design and results informed decision-making in setting up the UK National Breast Screening Programme.^14^

In this paper, we analyse breast cancer incidence and mortality data from both the Swedish Two-County Trial and the general female population in England before and after the inception of the screening programme. We derive simple deterministic estimates from both sources of the number of lives saved and numbers of cases overdiagnosed as a result of breast cancer screening.

## MATERIALS AND METHODS

The Swedish Two-County Trial has been described previously. Briefly, 55,985 women aged 40–74 were allocated to invitation to periodic mammographic screening (active study population, ASP) and 77,080 to no invitation (passive study population, PSP). Women in the ASP aged 40–49 at allocation were offered screening on average every 24 months. Women aged 50–74 were offered screening every 33 months. After 6–7 years, the PSP was invited to screening and the screening phase of the trial closed,^15^ but follow-up continued for deaths from breast cancers diagnosed during the screening phase. Subjects were randomized between 1977 and 1981 and spent an average of seven years in the screening phase of the trial. Thus, the screening in the trial took place between 1977 and 1988. In this paper, we have data on deaths till the end of 1998, a maximum of 21.5 years follow-up.

The UK National Breast Screening Programme was launched in 1988, although only approximately 2.5% of the target population was screened in that year. We therefore consider the screening epoch to be 1989 and thereafter. The programme was built up during the period 1989–1993. It offers three-yearly mammography screening. Originally, the age range intended was 50–64 years. In 2002–2004, this was expanded to 50–70 years, and a further expansion to the range 47–73 years is in progress. We have data by five-year age group and calendar year on breast cancer incidence between 1974 and 2003 and breast cancer mortality between 1974 and 2004, with the corresponding population denominators.

For the estimation of the absolute benefit of screening from the Two-County Trial, we used the same methodology as described previously,^1^ but applied it specifically to the age group 50–69 at randomization, to correspond approximately to the target group of the UK programme. Essentially we established the deficit in deaths from breast cancer in the study group compared with the control group and the numbers in the study group who were screened. Dividing the latter by the former gives an estimate of the number of women needed to screen to save one life. For the estimation of the benefit from the English Breast Cancer mortality data, we used Poisson regression to compare the difference in breast cancer mortality for ages 50–69 years between 1995 onwards and pre-1989 with that observed for other age groups. We calculated the absolute number of deaths prevented as the difference between those observed and those expected on the basis of the mortality changes in age groups <50 and 70 years or more. This is conservative because some of the deaths observed from 1995 onwards will be from tumours diagnosed before 1989, when there was no screening, and because some deaths in the period 1989–1994 will have been prevented by screening.^2^

The estimation of overdiagnosis is more complicated. In the Swedish Two-County Trial, we first estimated the expected incidence in the absence of screening as follows: from the incidence in the control group, we used Poisson regression to estimate the trend in incidence in the first six years of the trial, before any screening of the control group in this age group took place in order that our estimate of the underlying incidence trend was not contaminated by screening. From the trend we estimated the expected average incidence in year 4 after randomization, the midpoint of the screening phase of the trial.

Subjects in the ASP were invited to one prevalence screen and, on average, two incidence screens. Let *P* be the observed prevalence at first screen in the ASP, *Q* the observed prevalence at the first screen of the PSP at closure of the screening phase, *P*_T_ the unknown prevalence of true cases at first screen in the ASP, *P*_O_ the unknown prevalence of overdiagnosed cases at first screen in the ASP, *S*_T_ the rate of true cases at incidence screen, *S*_O_ the unknown rate of overdiagnosed cases at incidence screen, *I* the average annual incidence in the ASP during the screening phase, *I*_e_ the expected average annual incidence from PSP, *t* the relative incidence of breast cancer after seven years (i.e. at the end of the trial), taking into account age and time trends. As entry to the trial was in the period 1977–1981, this seven-year period pertains mostly to the early 1980s, where *a*_1_ is the proportion attending first screen of ASP and *a*_2_ is the proportion attending incidence screens of ASP.

The following equations will therefore hold:

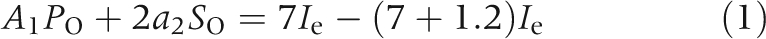


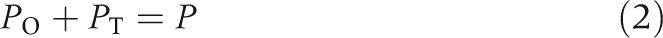


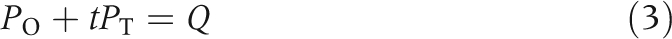


Here we are assuming that the same rate of overdiagnosed cases applies to the first screen of the ASP and PSP (approximately seven years older at the time of first screen), but that the true cases will reflect the trend with age and time. After excluding the prevalence screen, at which most length bias or overdiagnosis is likely to take place,^5,7,8^ the age at diagnosis in the ASP was 1.2 years on average younger than that in the PSP, suggesting a 1.2 year correction for overdiagnosis in equation (1). The exclusion of all of the prevalence screen cases in the estimation of lead time is likely to be conservative. *P*, *Q*, *I*, *a*_1_ and *a*_2_ were all directly observed from the trial data. *I*_e_ was observed in the PSP before the prevalence screen of this group and *t* was estimated from changes in breast cancer incidence by age and time from Swedish national statistics.^16,17^ In equation (1) we approximated the average expected incidence by the fitted incidence in year 4, the midpoint of the seven-year screening phase. Equations (1)–(3) were then solved for the three unknown quantities *P*_O_, *P*_T_ and *S*_O_.

For the estimation of overdiagnosis from the English incidence data, we analysed age groups <45, 45–49, 50–64, 65–69 and 70+ separately. We first estimated the log–linear trends in incidence from 1974 to 1988, before the screening programme started, using Poisson regression. We then projected these to estimate the expected incidence in 1989–2003. To adjust for any non-linear trends in log incidence, we re-estimated the expected incidence relative to age group <45, in which very little screening took place (some women receive their first invitation before age 50 but very few before age 45). This entailed dividing the expected numbers by the relative excess for the under 45 age group.

Overdiagnosis was calculated as the observed cases in the age groups 45–49 and 50–64, minus any deficit in age groups 65–69 and 70+, which is the sum of the observed minus expected figures for the four age groups.

## RESULTS

### Absolute numbers of lives saved

In the ASP of the Swedish Two-County Trial at ages 50–69 years, 90% attended the prevalence screen and 83% on average the two incidence screens.^15^ Table 1 shows breast cancer deaths, population sizes and numbers screened, with relative risk (RR), absolute number of breast cancer deaths prevented and the estimated number needed to screen to save one life. The RR of breast cancer death in the ASP compared with the PSP was 0.62 (95% CI 0.51–0.75). There were 201 breast cancer deaths in the ASP compared with 325 expected from the PSP (46,897 × 229/33,074). Thus, the estimated number of deaths prevented was 124, and the number needed to screen was 323 (40,060/124). This was the result of screening every 2–3 years for a period of seven years. With screening for 20 years at the same interval, one would anticipate 354 breast cancer deaths prevented (8.8 per thousand screened), and a number needed to screen of 113 to prevent one breast cancer death.

**Table 1:** Calculation of the number needed to screen to prevent one breast cancer death in the Swedish Two-County Trial, ages 50–69 years at randomization

Quantity	ASP	PSP
Number of subjects	46,897	33,074
Average number screened	40,060	–
Breast cancer deaths	201	229
Rate/1000	4.3	6.9
Deaths expected in ASP	325	–
Deaths avoided in ASP	124	–
Number needed to screen*	323	–

ASP, active study population; PSP, passive study population

*Number needed to screen to prevent one breast cancer death

**Figure 1:**
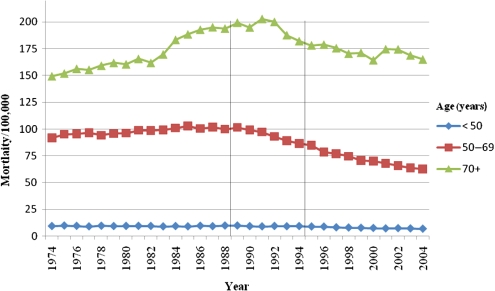
Breast cancer mortality in England 1974–2004

Figure 1 shows breast cancer mortality in England by time for age groups <50, 50–69 and 70+. There was a sharp decrease in mortality from the mid-1990s in the age group 50–69. In women aged <50, there was a lesser reduction in mortality, although this is difficult to see in the figure due to the rarity of breast cancer at younger ages. In the 70+ age group, there was a rise in mortality in the early 1990s then a fall back to 1980s' levels. Table 2 shows the mortality rates and RRs for the three age groups, and periods 1974–1988, 1989–1994 and 1995 onwards. The table also shows observed and expected numbers of deaths in 1995 onwards. Compared with other age groups there was a highly significant 28% reduction in breast cancer mortality in the age group invited to screening (RR 0.72, 95% CI 0.70–0.74, *P* < 0.001). The deficit in breast cancer deaths was 53,057–38,201 = 14,856. This corresponds to approximately 52 million person-years of screening, which implies 5.7 breast cancer deaths prevented for 1000 women screened for 20 years.

**Table 2:** Breast cancer mortality rates in England by age group, 1974–2004, with RRs, 95% CIs and observed/expected numbers of deaths in 1995–2004

Age group	Quantity	1974–1988	1989–1994	1995–2004	Observed (expected) number of deaths 1995–2004
<50	OR	1.00	0.99	0.82	12,623 (15,394)
	95% CI	(–)	(0.96–1.02)	(0.80–0.84)	
	Rate/100,000	9.4	9.3	7.7	
50–69	OR	1.00	0.97	0.73	38,201 (52,330)
	95% CI	(–)	(0.95–0.99)	(0.72–0.74)	
	Rate/100,000	97.7	94.6	71.5	
70+	OR	1.00	1.14	1.01	58,536 (57,956)
	95% CI	(–)	(1.12–1.16)	(0.99–1.03)	
	Rate/100,000	170.6	194.3	172.2	
50–69 (adjusted for other ages)*	OR	NA	0.84	0.72	38,201 (53,057)
	95% CI		(0.82–0.86)	(0.70–0.74)	

RR, relative risk; OR, odds ratio, NA, not applicable

*Adjusted RR for 1995–2004 is calculated as the unadjusted RR compared with 1974–1988 in the 50–69 age group, divided by the corresponding RR calculated for the other age groups

### Overdiagnosis

Average incidence in age group 50–69 in the PSP before screening was 0.0021.^15^ From Swedish national incidence rates published in *Cancer Incidence in Five Continents*,^16,17^ it was estimated as 1.35. Since *I* is the average incidence during the screening phase, we calculate it as the number of cases divided by seven times the average population during the screening phase. There were 46,897 women in the ASP in this age group, an average population during the screening phase of 45,155 and 911 cases diagnosed during the screening phase. This gave *I* = 911/(7 × 45,155) = 0.0029. Attendance at first screen of the ASP (*a*_1_) was 90% and the corresponding prevalence (*P*) was 0.0068. Average attendance at the two incidence screens in the ASP (*a*_2_) was 83%. Attendance at the first screen of the PSP at closure of the screening phase was 84% and prevalence (*Q*) was 0.0085. Equations (2) and (3) are therefore

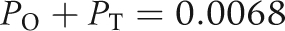

and

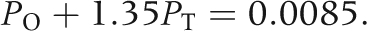


This gives *P*_T_ = 0.0049 and *P*_O_ = 0.0019. Substituting *P*_O_ in equation (1) gives

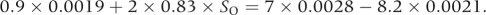


This in turn gives *S*_O_ = 0.0004, and a total overdiagnosis of 2.4 per 100c in the ASP, approximately 12% of cancers diagnosed. For per 1000 women screened seven times over 20 years, the expected number of overdiagnosed cases would be 1.9 + 6 × 0.4 = 4.3. With the estimated lives saved for this number of 8.8 per 1000, this means that for every two breast cancer deaths prevented, one might expect one overdiagnosed case. Put another way, for every 11 cases diagnosed, two lives will be saved, and one case will be overdiagnosed.

**Figure 2:**
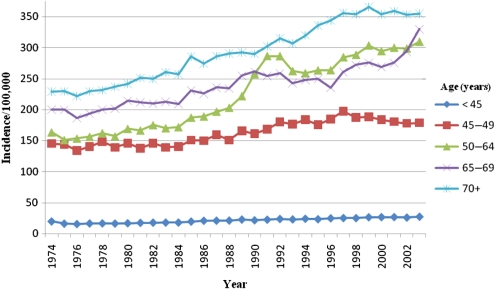
Breast cancer incidence in England 1974–2003

Figure 2 shows breast cancer incidence in England by age and time. Clearly, incidence was increasing prior to screening in all age groups and continued to do so thereafter, with a particular strong increase in age group 50–64 in the early years of the programme. A corresponding deficit can be seen shortly afterwards in the 65–69 age group, which also showed a sharp increase in 2002–2003 when the programme was expanded to include ages up to 70 years. At ages 70 years or more the incidence after 1996 was lower than that which would have been observed if prescreening trends had persisted. Table 3 shows the observed and expected numbers of cases by age group in years 1989–2003, standardized to the age group <45. There was an excess of a total of 25,042 tumours in age groups 45–64, but a deficit of 18,981 in ages 65 years and over. The net excess was therefore 6061 breast cancers. Taking this as the estimate of overdiagnosis from the 52 million person-years of screening, we estimate 2.3 cases overdiagnosed per 1000 women screened for 20 years. Thus, for every two breast cancer deaths prevented in the UK programme, we estimate that there is less than one overdiagnosed case. And for approximately every 28 cases diagnosed, 2.5 lives were saved and one case overdiagnosed. The larger number of tumours diagnosed per life saved and per overdiagnosed case is due to the higher incidence and greater survival in the late 1990s and early 21st century compared with the late 1970s and early 1980s.

**Table 3:** Observed cases of breast cancer in England 1989–2003, with expected cases calculated by extrapolation of pre-1988 trends and standardized to the age group <45 years

Age group	Observed (O) cases	Expected (E) cases*	O − E	RR (95% CI)
<45	54,780	54,780	0	1.00 (–)
45–49	42,962	40,467	2,495	1.06 (1.04–1.08)
50–64	168,253	145,706	22,547	1.15 (1.13–1.17)
65–69	47,044	49,844	−2,800	0.94 (0.92–0.96)
70+	168,656	184,837	−16,181	0.91 (0.90–0.92)

RR, relative risk

*Standardized to the age group <45

## DISCUSSION

Our estimates of the absolute benefits of screening were 8.8 and 5.7 breast cancer deaths prevented per 1000 women screened for 20 years from age 50, from the Swedish Two-County Trial and the Breast Screening Programme in England, respectively. The corresponding estimated overdiagnosed cases were 4.3 and 2.3 per 1000 over 20 years. This implies that in a cohort screened every three years for the 20 years from age 50 between 9% and 13% of cases diagnosed have their lives saved, and between 4% and 7% of cases are overdiagnosed. Thus, the benefits in terms of numbers of deaths prevented are around double the harms in terms of overdiagnosis. Analysis of both data-sets shows a substantial and significant reduction in breast cancer deaths in association with mammographic screening. This benefit is greater in absolute terms than the harm of overdiagnosis.

Our estimated benefit is somewhat larger than our previous estimates, which warrants some explanation.^1,2^ In this study we evaluated screening in the age group 50–69, whereas the previous estimates pertained to the age groups 40–69 or 40–74, in which both the absolute mortality from breast cancer and the effect of screening would be attenuated in comparison with 50–69. Our estimated absolute number of lives saved is considerably larger than that of a number of other studies.^6,18^ As has been pointed out in the past, however, the latter have suffered from confusion of actual screening with invitation to screening, confusion of follow-up periods with screening periods and exclusion of other relevant information.^12,19,20^

If we consider that in the unscreened age groups, crude rates of breast cancer incidence increased while mortality remained stable, it is clear that other factors such as improvements in treatment and earlier symptomatic diagnosis have also improved breast cancer's prognosis in the UK during the period under study. The reduction in mortality in the age group 50–69, however, indicates that the policy of screening has conferred a further 28% mortality reduction.

Our estimates of overdiagnosis are higher than our previous estimates from multistate modelling.^7^ As noted above, the figure from the Swedish Two-County Trial may be an overestimate of overdiagnosis. It is also possible that the multistate models somewhat underestimate overdiagnosis due to strong negative co-linearity with estimated screening sensitivity. Our present results lie between our previous estimates and those of the Advisory Committee on Breast Cancer Screening,^21^ which estimated that one in eight women who are routinely screened and are diagnosed with breast cancer have a breast cancer diagnosed, which would not have arisen in the absence of screening, and one in eight fewer such women would die of breast cancer.

These results are in stark contrast to those of Gøtzsche *et al.*,^6^ who have claimed that the overdiagnosed cases are 10 times more common than the breast cancer deaths prevented. The reason for this disagreement partly lies in the fact that our benefit is estimated directly from empirical data, and we have been careful to avoid confusing invitation to screening with actually receiving screening. It may also be due to the time frames in the two sets of estimates. Gøtzsche and colleagues base their estimates on cases and deaths occurring during a 10-year screening period. Much of the benefit of screening in a 10-year period will actually be observed after that period, and excess cases occurring during that period may be compensated for by a future deficit in incidence. This emphasizes that to estimate absolute benefits and harms, long periods of follow-up are necessary. Interestingly, Jørgensen and Gøtzsche^22^ recently estimated a 57% excess incidence in the screening age range in the first seven years of the UK programme and interpreted this as entirely due to overdiagnosis. This in turn would imply that 36% of the tumours diagnosed in this age group and period were overdiagnosed. Since only 37% of breast cancers were diagnosed by screening during this period,^23^ this would mean that almost 100% of screen-detected cancers would never have arisen during the lifetimes of the patients, which is an absurd and frankly incredible conclusion. We obtained less extreme and more credible estimates by having a longer observation period, incorporating the deficit in incidence after the upper age limit for screening and taking fuller account of other changes in incidence occurring independently of screening.

We have deliberately derived simple age-specific estimates from the English incidence and mortality rates. More complex age–period–cohort analyses might yield different estimates, although they would also have ambiguities of interpretation, due to the possibility of over-adjustment of potential effects of screening on incidence or mortality. At any rate, our estimates from both service screening and a major randomized trial show that there is a worthwhile benefit of mammography in terms of lives saved, and that this significantly exceeds any harm in the form of overdiagnosis that may occur.
